# A new biomechanical classification system for split fractures of the humeral greater tuberosity: guidelines for surgical treatment

**DOI:** 10.1186/s13018-021-02839-y

**Published:** 2021-11-24

**Authors:** Gang Liu, Xiaoguang Guo, Qian Zhao, Bo Qin, Junjie Lu, Dingsu Bao, Shijie Fu

**Affiliations:** 1 Department of Orthopedics, Affiliated Traditional Chinese Medicine Hospital of Southwest Medical University, Luzhou, Sichuan China; 2 Center for Orthopedic Diseases Research, Affiliated Traditional Chinese Medicine Hospital of Southwest Medical University, Luzhou, Sichuan China; 3 Expert Workstation in Luzhou, Luzhou, Sichuan China; 4 Clinical Base of Affiliated Traditional Chinese Medicine Hospital of Southwest Medical University, Guangdong Province Medical 3D Printing Application Transformation Engineering Technology Research Center, Luzhou, Sichuan China; 5 Department of Orthopedics, YiXing Traditional Chinese Medicine Hospital, Yixing, Jiangsu China; 6 Department of Breast Surgery, Luzhou Hospital of Traditional Chinese Medicine, Luzhou, Sichuan China

**Keywords:** Humeral greater tuberosity, Split fracture, Biomechanical classification, Rotator cuff tear, Surgical technique

## Abstract

**Background:**

Split fractures of the humeral greater tuberosity (HGT) are common injuries. Although there are numerous surgical treatments for these fractures, no classification system combining clinical and biomechanical characteristics has been presented to guide the choice of fixation method.

**Methods:**

We created a standardised fracture of the HGT in 24 formalin-fixed cadavers. Six were left as single-fragment fractures (Group A), six were further prepared to create single-fragment with medium size full-thickness rotator cuff tear (FT-RCT) fractures (Group B), six were cut to create multi-fragment fractures (Group C), and six were cut to create multi-fragment with FT-RCT fractures (Group D). Each specimen was fixed with a shortened proximal humeral internal locking system (PHILOS) plate. The fixed fractures were subjected to load and load-to-failure tests and the differences between groups analysed.

**Results:**

The mean load-to-failure values were significantly different between groups (Group A, 446.83 ± 38.98 N; Group B, 384.17 ± 36.15 N; Group C, 317.17 ± 23.32 N and Group D, 266.83 ± 37.65 N, *P* < 0.05). The load-to-failure values for fractures with a greater tuberosity displacement of 10 mm were significantly different between each group (Group A, 194.00 ± 29.23 N; Group B, 157.00 ± 29.97 N; Group C, 109.00 ± 17.64 N and Group D, 79.67.83 ± 15.50 N; *P* < 0.05). These findings indicate that fractures with a displacement of 10 mm have different characteristics and should be considered separately from other HGT fractures when deciding surgical treatment.

**Conclusions:**

Biomechanical classification of split fractures of the HGT is a reliable method of categorising these fractures in order to decide surgical treatment. Our findings and proposed system will be a useful to guide the choice of surgical technique for the treatment of fractures of the HGT.

## Background

The humeral greater tuberosity (HGT) is the attachment point of the rotator cuff, which is the axis of the shoulder and plays an important role in shoulder movement. Proximal humeral fractures (PHFs) are the third most common fractures in elderly individuals, accounting for 5% of all fractures among such patients [1, 2]. In contrast, fractures of the HGT occur more frequently in younger patients with strong bones following high-velocity trauma. These fractures are common and account for up to 20% of all PHFs [3, 4].

**Fig. 1:**
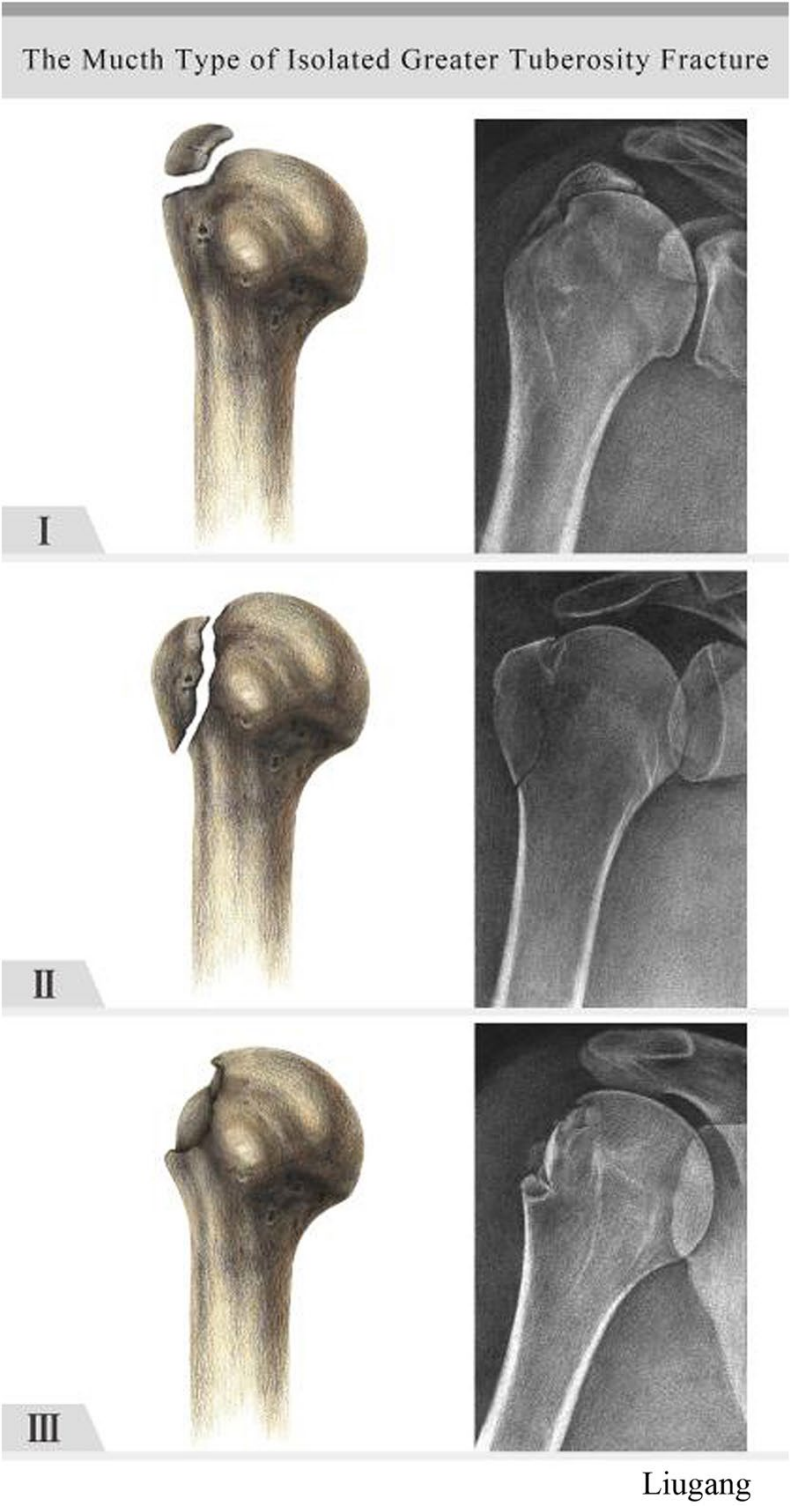
Morphological classification of fractures of the HGT. Type I avulsion fractures exhibit small fragments of bone with a horizontal fracture line. Type II split fractures exhibit one large fragment with a vertical fracture line. Type III depressed fractures exhibit an inferiorly displaced fragment

There are three classification systems that are routinely used to assess GT fractures; namely, the Neer, AO and morphological classification systems [5–7]. The Neer system classifies PHFs into four categories based on location: the GT, the lesser tuberosity, the humeral head or the humeral shaft if there is displacement of > 1 cm or angulation of > 45. However, in 2005, Kim reported that isolated GT fractures have different characteristics to other PHFs; thus, the treatment and classification of these fractures should be different to that of other PHFs [8]. The AO system classifies GT fractures as non-displaced, displaced or associated with shoulder dislocation. Recent studies on split fracture have focused mostly on morphological classification. In 2014, Mutch proposed a classification system dividing GT fractures into three types: avulsion, split or depressed. Split fractures of the GT (Fig. 1) are the most common, accounting for 41% of all GT fractures. Yet, there is currently no classification system for split fractures of the HGT with rotator cuff tear (RCT) which considers the number of fragments.

Coexisting soft tissue lesions of greater tuberosity fractures were discussed in several reports as being possible indications for surgical treatment and that might result in persistent late pain, shoulder dysfunction [9, 10]. Early in 1996, Gary reported a non-union of the greater tuberosity fracture concomitant with full-thickness rotator cuff tear and it was successfully treated with arthroscopy [11]. Gumina described 24 patients with missed greater tuberosity fractures, of which 11 (45.8%) had rotator cuff tears with the use of magnetic resonance imaging (MRI) for detecting soft tissue pathologies [12]. Moreover, Eran Maman reported rotator cuff tears were the most commonly diagnosed pathology of coexisting lesions with GT fractures, of which the supraspinatus was the most frequently involved tendon (36% of all pathologies). He believes that it is important to identify and repair pathologies concomitant with GT fractures [13].

**Fig. 2:**
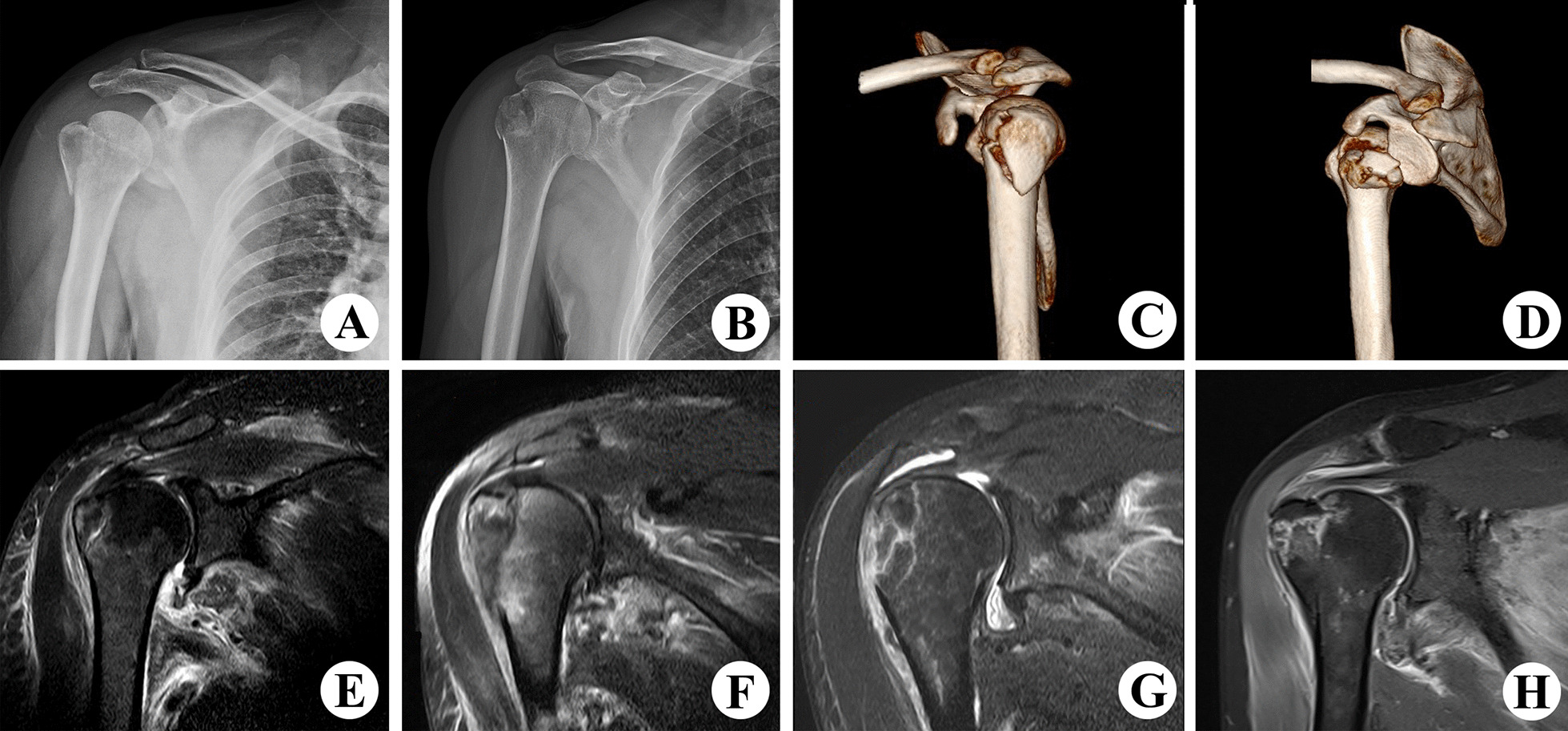
Clinical imaging findings of fractures of the humeral greater tuberosity. **A** X-ray image of a single-fragment fracture. **B** X-ray image of a multi-fragment fracture. **C** Computed tomography scan of a single-fragment fracture. **D** Computed tomography scan of a multi-fragment fracture. **E** Magnetic resonance image of a single-fragment fracture. **F** Magnetic resonance image of a single-fragment fracture with rotator cuff tear. **G** Magnetic resonance image of a multi-fragment fracture without rotator cuff tear. **H** Magnetic resonance image of a multi-fragment fracture with rotator cuff tear

**Fig. 3:**
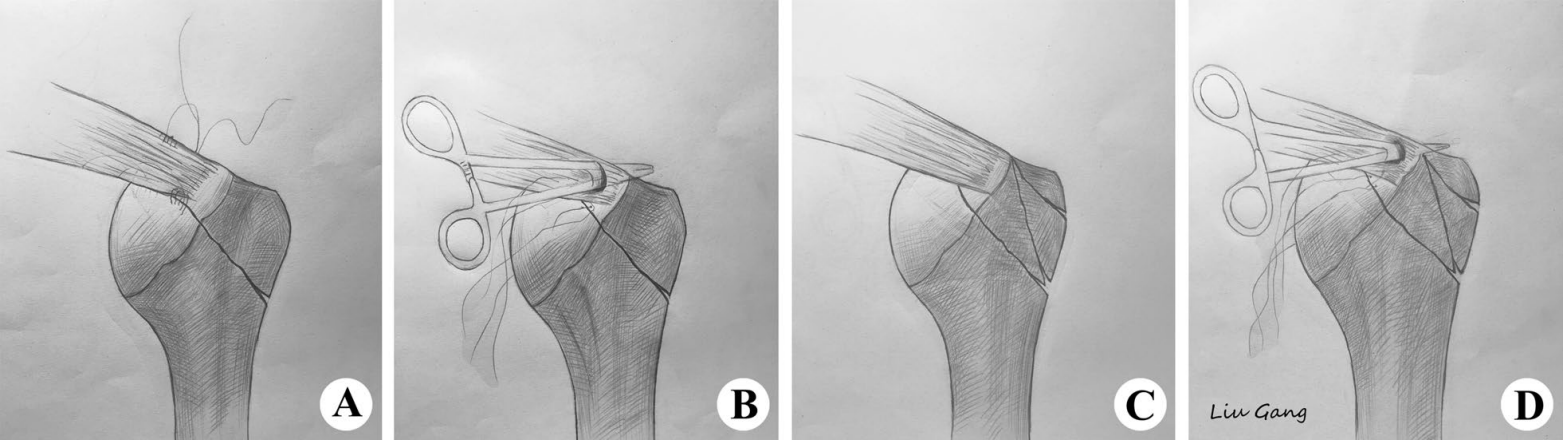
Sketch models of the four types of fracture of the humeral greater tuberosity. **A** Type I, single-fragment fracture. **B** Type II, single-fragment fracture with medium size full-thickness rotator cuff tear (FT-RCT). **C** Type III, multi-fragment fracture. **D** Type IV, multi-fragment fracture with medium size FT-RCT

Despite their high prevalence, coexisting Lesions have largely been ignored. There have been few clinical guidelines on comminuted fractures or RCT in the context of fractures of the HGT. According to our clinical data findings from January 2010 to January 2018 (Fig. 2), such injuries have different shoulder function outcomes; particularly fragmented fractures or those combined with RCT. The present study sought to address the following questions: are there any significant differences between fractures that are categorised according to specific clinical features? What are the optimal treatments when these differences are considered? We created four cadaver models (Fig. 3), evaluated whether there are significant differences between the models and identified optimal operative treatments for each.

The aims of this study were to: (i) create four cadaver models from our long-term clinical data (Fig. 3); (ii) identify the different characteristics of each model and (iii) identify the optimal treatment for different types of fractures. The primary hypothesis was that biomechanical classification of fractures of the HGT is a reliable system and can be used to guide the choice of surgical technique.

## Methods

### Specimen selection and preparation

This study was conducted in the Biomechanical and Anatomy Laboratory of the South-west Medical University, Sichuan, China. We selected donors whose family had given written consent for the donation of their to science. Inclusion criteria were cadavers from Chinese patients that had: (i) been soaking in the same formalin mixture for 6 months, (ii) full-grown and normal shoulder joints, (iii) no history of previous shoulder operations, (iv) no history and/or signs of previous fracture, (v) a cadaveric age of less than 60 years and (vi) normal BMD. Exclusion criteria were: (i) history of diabetes or smoking, (ii) history of soft tissue injury in the AC or shoulder joint (e.g. osteoarthritis, shoulder instability or RCT) and (iii) incomplete specimens.

Bone density was assessed by X-ray (OSTEOCORE-3; Golden, China), and BMD was compared between the four groups using one-way analysis of variance (ANOVA) to ensure that there were no significant differences in BMD, which could affect the biomechanical results. Finally, 24 formalin-fixed (35% formaldehyde in alcohol; Da-pin chemical industry, Guangzhou, China) cadaver shoulder specimens (16 right and 8 left; 16 men and 8 women) were used for this study.

**Fig. 4:**
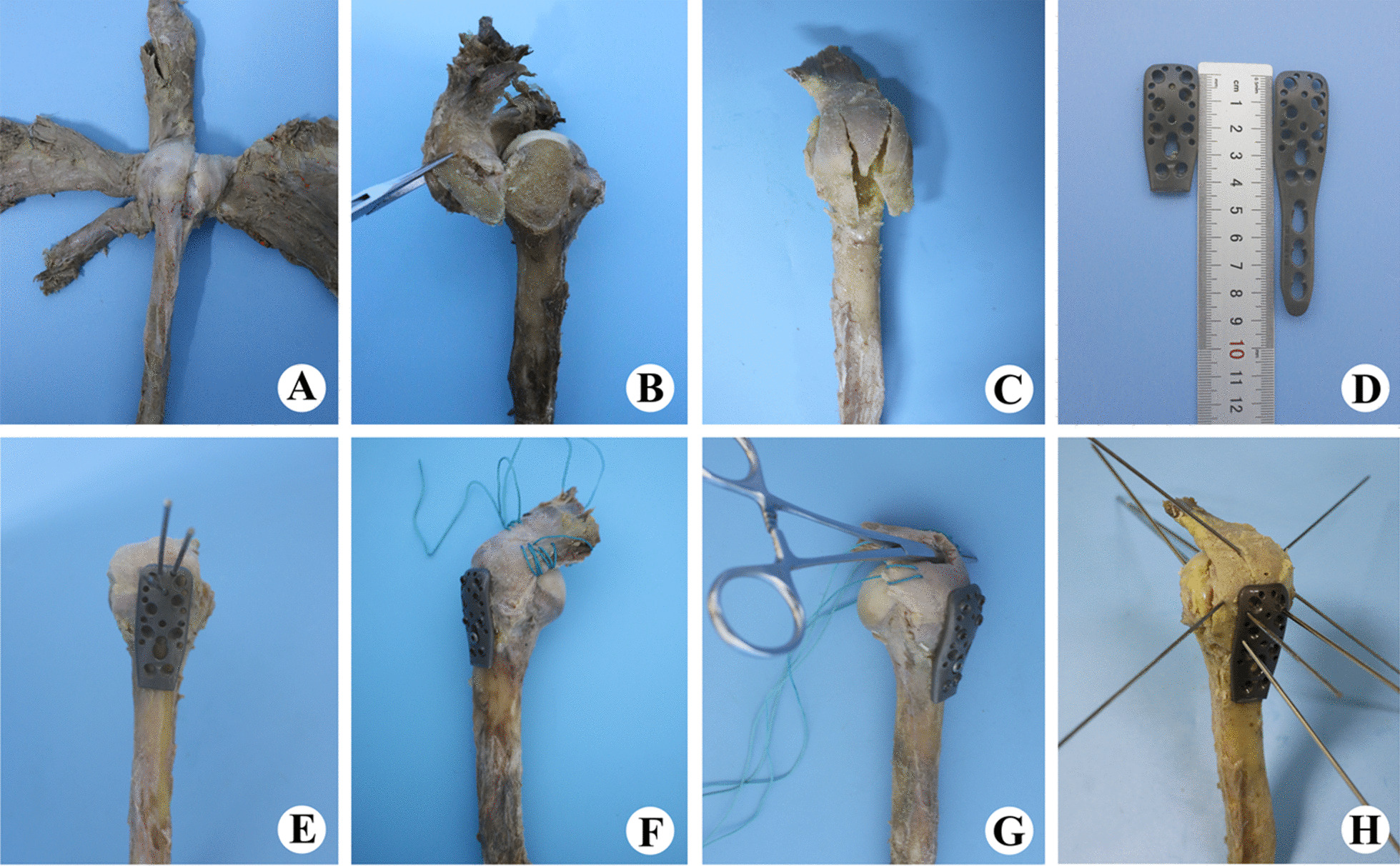
Diagram showing the preparation of specimen models. **A** The whole humerus and rotator cuff tendon were retained. **B** A standardised greater tuberosity fracture. **C** The single-fragment fracture was cut into multiple fragments. **D** Comparison of the PHILOS and shortened PHILOS plates. **E** Standardised fixation of single-fragment fractures. **F** Both sides of the supraspinatus were sutured using Ethicon 5# Johnson suture material. **G** A medium size, full-thickness rotator cuff tear was created. **H** Fixation of multi-fragment fractures

To prepare the specimens, we resected all soft tissue from the scapula and humerus, retaining only the whole humerus and rotator cuff tendon (Fig. 4A). Parts of the supraspinatus and tendon of > 5 cm in length were preserved at the bony insertion. We created a standardised Type I GT fracture at an angle of 50° to the shaft of the humerus using a thin-blade reciprocating saw (Guoke, China) as previously described [14] (Fig. 4B). Six models were left as Type I fractures (Group A), while 12 were further cut on both sides of the single-fragment to create multi-fragment fractures (Fig. 4C). Finally, we chose six single- and six multi-fragment models to be used to create models with medium size (i.e. 1–3 cm according to the Cofield Classification), full-thickness (FT) RCT [15]. The thickness and width of the supraspinatus were measured using digital callipers, and the supraspinatus footprint area was marked and measured.

### Fixation configurations

We chose to modify a small locking plate (a shortened PHILOS plate, HS-A-BU0173-029, China; Fig. 4D) to fix the fractures, in line with guidelines for the surgical treatment of displaced fractures of the HGT [16]. All operations were performed by the senior author (Shijie-Fu). The shortened PHILOS plate was prepared by cutting off the three-hole screw on the plate shaft using strong scissors (Guoke, China). Then, fragments were accurately fixed using K-wires. The PHILOS plate was fixed at 5 mm below the top of the GT and the medial intertubercular sulcus, according to the protocol of Ali-jabran [17]. We fixed the medium size (1.5-cm) FT-RCT of Groups B and D by suturing both sides of the supraspinatus (Fig. 4F) using Ethicon 5#, a high-polymer polyethylene (Johnson, USA).

### Load test

**Fig. 5:**
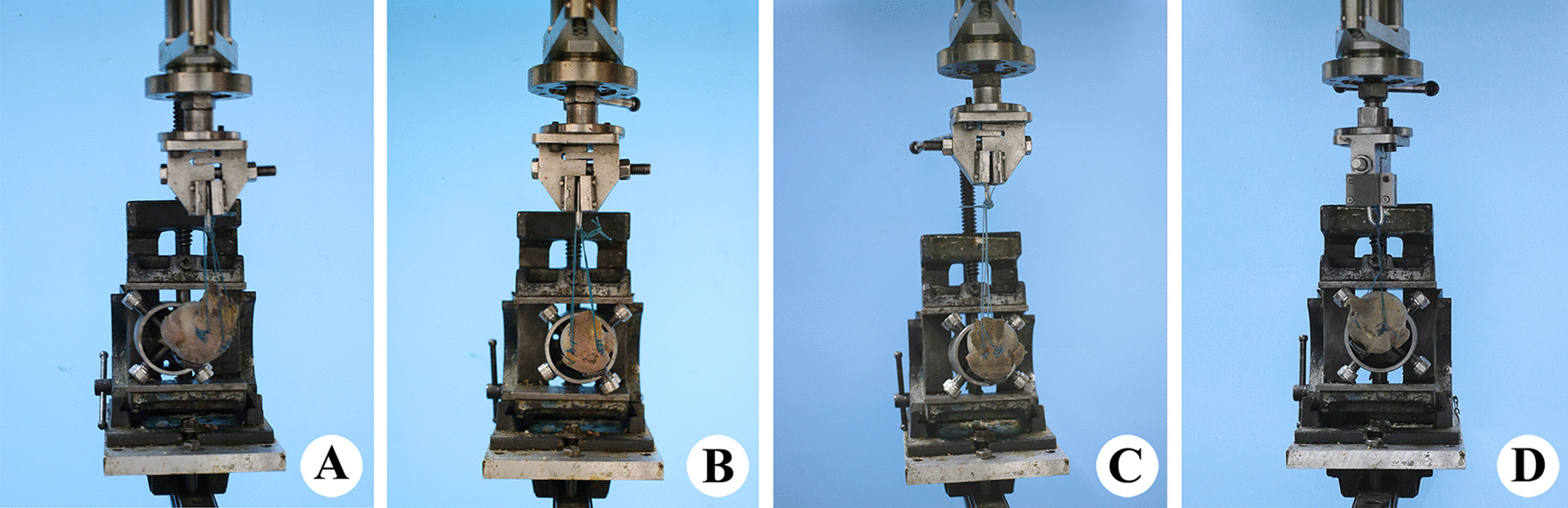
Diagrams of the load and load-to-failure tests. **A** Group A, single-fragment fracture. **B** Group B, single-fragment fracture with medium size full-thickness rotator cuff tear (FT-RCT). **C** Group C, multi-fragment fracture. **D** Group D, multi-fragment fracture with medium size FT-RCT

Specimens were placed in a special clamp (Fig. 5) to ensure stability during the load test. All tests were performed at room temperature, and the surface of the prepared-modal was kept constantly moist with isotonic saline. One side of the specimen was fixed to the biomechanical testing machine (Bose Electro Force 3520-AT, USA), and the other was fixed to the upper part of the testing machine (Fig. 5). During mechanical tests, the supraspinatus was fixed at an angle of 90° abduction. A superior preload of 50 N was then applied out to assess the time effect, stress relaxation and stability of specimen fixation. The distance at 5 N was set as the initial reference (starting point). The electrodynamic testing machine applied a load at a constant speed of 5 N/s. The load test was repeated 10 times with intervals of 3 min to avoid stress fatigue.

### Load-to-failure test

Tests to assess ultimate failure load (N) were performed at a constant speed of 1 mm/min in the superior-inferior direction, and the mode of failure was recorded. We also recorded results for fractures with 3, 5 and 10-mm displacement of the HGT. Failure was defined as RCT rupture, internal fixation failure or complete dislocation of HGT.

### Statistical analysis

The Statistical Package for the Social Sciences (SPSS) 19.0 software (Chicago, IL, USA) was used for all statistical analyses. All data are presented as mean ± standard deviation ($\overline{x} \pm s$). Homogeneity of variance was evaluated using the Shapiro–Wilk test. One-way ANOVA was used for multiple comparisons between groups when the variances were homogeneous. A significance level of *P* < 0.05 was accepted as statistically significant.

## Results

### Specimens and basic physical properties

The mean age of the cadavers at the time of death was 43.5 (range: 29–52) years. There were no significant differences in BMD, supraspinatus thickness, tendon width, footprint thickness or footprint width between the groups (Table 1).

**Table 1:** Basic physical properties of the cadaver specimens were as follows

Physical properties	Group A	Group B	Group C	Group D	*P*
BMD, g/cm^2^	0.52 ± 0.03	0.52 ± 0.05	0.51 ± 0.06	0.52 ± 0.05	0.26
SS thickness (mm)	5.25 ± 0.42	5.37 ± 0.57	5.01 ± 0.67	5.34 ± 0.37	0.50
SS width (mm)	24.23 ± 2.53	24.15 ± 2.52	23.73 ± 2.37	24.08 ± 2.26	0.92
Footprint length (mm)	12.36 ± 1.88	12.26 ± 1.41	12.42 ± 1.19	11.96 ± 1.51	0.65
Footprint width (mm)	23.01 ± 1.52	22.59 ± 1.78	22.35 ± 1.49	23.43 ± 2.01	0.54

Group A (n) = B = C = D = 6, *BMD* bone mineral density, *SS* supraspinatus

### Displacement of fractures of the humeral greater tuberosity

Results relating to 3-, 5- and 10-mm displacement of fractures of the HGT are summarised in Table 2. A steady but significant decrease was observed among the fractures with 10 mm displacement from Groups A to D. However, there were no statistically significant differences between the fractures with 10 mm displacement and those with 3- or 5-mm displacement within any group.

**Table 2:** The findings of 3 mm, 5 mm and 10 mm HGT displacements

Displacement (mm)	Group A (N)	Group B (N)	Group C (N)	Group D (N)
3	33.50 ± 3.39^bc^	31.17 ± 6.24	27.17 ± 5.34	27.00 ± 3.74
5	80.17 ± 10.01^bc^	66.83 ± 17.80^bc^	45.00 ± 5.83	41.50 ± 8.19
10	194.00 ± 29.23^abc^	157.00 ± 29.97^bc^	109.00 ± 17.64^c^	79.67 ± 15.50

Each group contained six specimens, a: versus Group B, b: versus Group C, c: versus Group D (*P* < 0.05)

### Load-to-failure

**Fig. 6:**
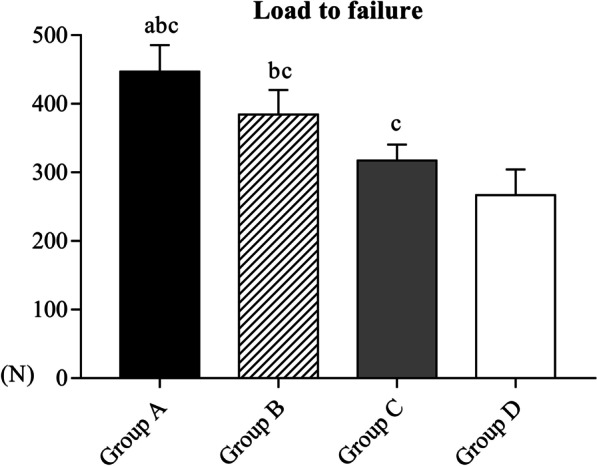
Results of the load-to-failure test. Notes: a versus Group B; b versus Group C; c versus Group D (*P* < 0.05)

Of the four groups, Group A had the highest mean load-to-failure value (446.83 ± 38.98 N), and the mean value was significantly different between each group (Group B, Type II fracture, 384.17 ± 36.15 N; Group C, Type III fracture, 317.17 ± 23.32 N and Group D, Type IV fracture, 266.83 ± 37.65 N) (Fig. 6). The mode of failure was fracture at the humeral surgical neck in six cases of Group A. Failure was humeral surgical neck fracture in five cases and GT fragment displacement in one case of Group B. In Group C, the mode of failure was GT fragment pulled out in two cases, surgical neck fracture in three cases and anatomic neck fracture in one. In Group D, one case failed due to rotator cuff rupture, two due to GT- fragment pulled out and three due to surgical neck fracture.

## Discussion

Our study demonstrates that there are significant differences between the characteristics of HGT fractures with 10-, 5- and 3-mm displacements in terms of load-to-failure. This suggests that different surgical approaches should be considered depending on the magnitude of displacement in such fractures. We have also shown that biomechanical classification of split fractures of HGT into Type I (single-fragment), Type II (single-fragment with medium size FT-RCT), Type III (multi-fragment) and Type IV (multi-fragment with medium size FT-RCT) fractures is reliable and can be used to guide the choice of surgical technique, thus confirming our primary hypothesis. To the best of our knowledge, this is the first study to describe the biomechanical differences between split fractures of the HGT using long-term clinical data.

Split fractures of the HGT involve lesions of the bone and rotator cuff matter. The present study revealed that Type IV fractures are most susceptible to failure following fixation with a shortened PHILOS plate. This is an important finding as it suggests that the number of fragments or inclusion of an RCT affects the final result of surgery. Furthermore, Type IV fractures were more likely to exhibit 10-mm displacement. This highlights the necessity for clinicians to be aware of associated symptoms and to fix the RCT with a suture anchor at the same time as fixing the fracture. This information will enable improved preoperative planning and results in terms of shoulder function. Interestingly, there were no significant differences between groups among fractures with 3- or 5-mm displacement. This may be due to the use of a shortened PHILOS plate, which is a firm fixation. Significant differences between groups might become apparent if the fractures were fixed through fixation using double-row sutures or the suture bridge technique. Future studies investigating the implications of different surgical techniques are warranted to evaluate the outcomes of different approaches.

There have been numerous studies focusing on indications for surgical treatment and fixation technique; however, only a few have focused on injuries involving both fragmentation and RCT [18–21]. An increasing number of studies have been published reporting injuries of the GT [22–24]; however, to the best of our knowledge, there have been none comparing single- and multi-fragment fractures. Some studies have examined associated injuries such as Bankart lesions, RCT and superior labral tear from anterior and posterior (SLAP) lesions in the context of these fractures [25]. Locking plate fixation provides superior fixation for split-type GT fractures compared with tension bands or double-row suture bridges. Therefore, we choose to use a shortened PHILOS plate for fixation in the present study. Previous studies have reported load-to-failure values of 842 or 1054 N, considerably higher than the results of the present study [24]. This is likely due to the fact that we used formalin-fixed cadaver shoulder specimens.

Three main techniques of surgical fixation have been described for HGT fractures, with different techniques being more suitable for different types of fracture. Our study provides a system with which to classify fractures of the HGT and guide the choice of fixation technique. The specific recommendations that we propose are as follows: (i) Type I (single-fragment) fractures should be fixed using compression screws, which are inexpensive and efficient and have been shown to have favourable results through biomechanical studies. This is a useful approach for areas in which patients cannot afford high medical expenses and/or have insufficient health insurance and is also beneficial because the insertion angle can be adapted to increase biomechanical strength following fixation of osteoporotic fractures; a subject which warrants further study. However, compression screws may cause damage to fracture fragments [7, 16, 24]. (ii) Type II (single-fragment with medium size RCT) fractures should be fixed using screws combined with suture anchors under arthroscopic guidance. This method is widely used to treat PHFs as the tendon-bone interface fragment is fixed and satisfactory clinical results can be achieved [16, 18]. (iii) Type III (multi-fragment) fractures should be fixed using a suture bridge or small locking plate to provide stable fixation and early return to function. This surgical technique is simple and efficient [26–28]. (iv) Type IV (multi-fragment with medium size RCT) fractures should be fixed using a small locking plate augmented with suture anchors via a mini-open deltoid-split approach [13, 16, 23, 24, 29].

The present study has some limitations which should be acknowledged. Firstly, we did not justify the use of this method of 3D reconstruction compared to other methods in patient with GT fracture. Due to a lack of human samples, we used 24 formalin-fixed specimens. Future studies should be carried out using fresh-frozen human cadaveric specimens. Secondly, the clinical models were not assessed by computed tomography scan or magnetic resonance imaging, which would have provided useful information. Thirdly, with the limited number of available cadaver specimens, we were unable to evaluate all known fixation techniques. With more specimens, further fixation techniques and the application of multi-planar ultimate loads could be evaluated.

## Conclusions

The present study demonstrates that biomechanical classification of split fractures of the HGT is a reliable classification system. Although numerous surgical treatments for these fractures have been described, there is no gold standard in terms of treatment for this type of fracture. Therefore, our classification system will be a useful guide to enable surgeons to select an appropriate surgical technique. In the future work, we will validate this study with 3D Simulation (Finite element analysis).

## Data Availability

All data generated or analysed during this study are included in this published article and its supplementary information files.

## Abbreviations

HGT: Humeral greater tuberosity
FT-RCT: Full-thickness rotator cuff tear
PHILOS: Proximal humeral internal locking system
PHFs: Proximal humeral fractures
